# Resting Frontal Asymmetry and Reward Sensitivity Theory Motivational Traits

**DOI:** 10.1038/s41598-018-31404-7

**Published:** 2018-09-03

**Authors:** Vilfredo De Pascalis, Kathrin Sommer, Paolo Scacchia

**Affiliations:** Department of Psychology, La Sapienza University of Rome, Rome, Italy

## Abstract

The revised reinforcement sensitivity theory (rRST) of personality has conceptualized three main systems: the behavioural approach system (BAS), behavioural inhibition system (BIS), and fight-flight-freeze system (FFFS). Research links greater relative left-frontal activity with BAS-related tendencies and impulsivity and greater relative right-frontal activity with “withdrawal” motivation that included both BIS and FFFS. Although rRST has addressed the separation of FFFS and BIS, much of personality neuroscience research does not indicate which system is related to right frontal activity. We administered the Reinforcement Sensitivity Theory of Personality Questionnaire (RST-PQ) to measure the BAS and its facets (goal-drive persistence, reward interest, reward reactivity, and impulsivity), BIS, and the withdrawal FFFS. We examined the association of RST-PQ traits with resting electroencephalogram (EEG) alpha-asymmetry in female participants (N = 162) by considering the influence of experimenter’s gender. In the total group, that included two subgroups with experimenters of different gender, BAS-impulsivity was related to greater left- than right-frontal activity, and FFFS, but not BIS, was related to greater relative right-frontocentral activity. These associations remained significant for the subgroup with a young same-sex experimenter, but not with opposite-sex experimenter.

## Introduction

The original Reward Sensitivity Theory (RST) of personality^1,2^ emphasized only two of the systems underlying human behaviour: the behavioural approach system (BAS) and the behavioural inhibition system (BIS). The BAS was conceptualized to embody approach- and reward-related responses to signals of reward. The BIS was thought to control goal-directed behaviour to aversive stimuli and signals of punishment and was described as the system of anxiety. This theory also mentioned a less delineated fight-flight system (FFS) that was responsible for fear. In this original view, what is less apparent is the hidden complexity in and between these systems, which makes any attempt to provide a psychometric description of them far from ordinary and prone to confusion. Thus, the revised RST rRST^3,4^; developed the FFS further into a fight-flight-freeze system (FFFS), which was clearly distinct from the BIS and was primarily responsible for controlling all forms of aversive stimuli, including frustrating nonreward, fighting threats, fleeing in active avoidance, or freezing to avoid predators’ interest. In the rRST, the BIS becomes responsible for resolving all forms of goal conflict, including the co-activation of the BAS and the FFFS. Although the newer classes of RST measures have conceptualized the separation of the FFFS and the BIS, most of them still conceive of the BAS as a unitary dimension. However, there is compelling evidence that the BAS is multidimensional, both on the basis of empirical evidence^5,6^ and on theoretical grounds^4,7^. According to Corr^4^, behavioural activation processes consist of a number of cognitive operations involving working memory and executive controls, such as identifying the biological reinforcer, planning behaviour, executing the plan, and physically grasping the final biological reinforcer^4,8^. A popular RST questionnaire, showing excellent reliability and validity data, is Carver and White^5^ BIS/BAS scales. This instrument includes one scale to measure BIS and three scales to measure BAS functioning (Drive, Reward Responsiveness, and Fun Seeking). The BIS/BAS questionnaire has been widely employed in electroencephalography (EEG) research, using resting EEG alpha activity to relate the relative right-frontal activation with BIS scores and the relative left-frontal activation with BAS scores.

### BIS/BAS and EEG alpha asymmetry

Alpha band power is inversely related to cortical activation, since alpha frequency desynchronizes in response to activation^9,10^. The relative activation in homologous areas of the left and right hemispheres is typically calculated by subtracting the resting EEG alpha power of the cortical area of interest in the left hemisphere from that of the right hemisphere^11^. Original research on the BIS/BAS and resting alpha asymmetry found inconsistent results^12^. Two original studies observed that individual differences in the BAS related to greater left- than right-frontal activation at resting baseline^13,14^. One of these studies also reported that individual differences in the BIS related to greater right- than left-frontal alpha activity during baseline^14^, whereas the other found no relationship between the BIS and asymmetric frontal activity^13^. Psychobiological models of approach, avoidance, and inhibition systems have mainly highlighted the BAS and FFFS systems as major descriptors of personality and temperament^15–22^. Research investigating asymmetrical activation of the frontal cortex with approach/avoidance behaviour has shown that approach/BAS is associated with greater relative resting left-frontal activity at baseline^13,21^, e.g.^23–29^, for review see^30^. Although the relationship between the BAS and left-frontal activity appeared to be more replicable than the relationship between the BIS and right-frontal activity, a greater relative right-frontal cortical activation is found to be associated with conflict-related avoidance/BIS, although this support has received less empirical attention^14,31–33^. However, despite evidence linking trait frontal activity with the FFFS, many studies have failed to replicate the finding that withdrawal is associated with greater trait or state right-frontal activity^34^.

From the perspective of RST, the major problem with BIS/BAS scales is the lack of separation of the FFFS and the BIS, which may account for inconsistent findings in past research when relating the BIS scale to resting frontal alpha activity. Recent study findings suggest that these mixed results may be due to items in the BIS scale assessing both the FFFS and revised-BIS. In particular, Neal and Gable^33^, deriving BIS and FFFS subscales from the original Carver and White^5^ BIS/BAS scales, demonstrated that the BIS subscale, but not the FFFS, related to greater relative right-frontal activity, while a measure of impulsivity related to less relative right-frontal activity.

Very recently, Corr and Cooper^35^ developed the Reinforcement Sensitivity Theory Personality Questionnaire (RST-PQ) to facilitate future research, specifically on rRST and, in general, on approach-avoidance theories of personality. This questionnaire employed theoretically motivated thematic facets to cover the defensive space, comprising the FFFS (Flight, Freeze, and Active Avoidance) and the BIS (Motor Planning Interruption, Worry, Obsessive Thoughts, and Behavioural Disengagement), allowing the important separation between these two systems and the important distinction of reward sensitivity and impulsivity. The four sub-scales of the BAS – Reward Interest (BAS-RI), Goal-Drive Persistence (BAS-GDP), Reward Reactivity (BAS-RR), and Impulsivity (BAS-I) – allow this tool to test the instrument’s unique predictive power and redundancies. The first two BAS facets characterize the early stages of approach (“anticipatory pleasure” or “hope”), the latter two the behavioural and emotional “excitement pleasure attack” as the final biological reinforcer is reached. According to Corr^36^, this four-factor model updates Carver and White^5^ three-factor model of trait approach behaviour. According to Corr^37^ it is especially important to separate BAS-RI, BAS-RR and BAS-I. This distinction is since the first facet concerns the individual disposition to identify the biological reinforcer, the second the individual differences in emotional response to reward, and the third is tailored to reflect the final action for the capture of the desired object^4^.

In the present study, we administered the RST-PQ to a group of female participants to examine the link between the BIS and FFFS scales and asymmetry in frontal alpha activation.

Based on a body of previous research linking personality traits of regulatory control to right-frontal activity^33,34^, we expected that the BIS, as measured by the RST-PQ, would be related to relative right-frontal activity. In addition, considering the recent findings of a link between impulsivity and less relative right-frontal activity^33^ we predicted that greater BAS-I would be related to enhanced left-frontal activity.

A meta-analysis of studies examining the relation of the BAS with frontal alpha asymmetry yielded that this association is less robust than usually assumed^38^. The authors speculated that a link between alpha-asymmetry and the BAS emerged only among male participants when the experimenter was an attractive female. This hypothesis was also advanced by Schneider and collaborators, who failed to find a significant correlation between alpha asymmetry and personality for adolescents^39^. It also seems consistent with the significant association of BAS with frontal alpha asymmetry found in a study of our own among female participants with a male experimenter^25^. These observations call into question the hypothesis that the BAS is driven by patterns of resting cortical asymmetry and suggest the capability model of frontal EEG asymmetry and personality^40^ to account for inconsistent EEG asymmetry findings across studies. This view suggests that individual differences in affective tendencies will emerge as the product of the interaction between the innate capabilities of the individual and the situational context^41^. Thus, given the above-mentioned evidence, a further aim of the present study was to test the hypothesis that the situation in which female participants were undergoing a resting EEG assessment by a young male experimenter (i.e., in a situation stimulating an approach motivation) would uncover a robust association of approach-oriented traits with greater resting EEG alpha asymmetry. In contrast, when female participants were interacting with a young female experimenter, we expected an association of withdrawal motivation traits with resting EEG asymmetry in favour of the right frontal cortex.

## Results

Correlations among RST-PQ personality traits and descriptive statistics are shown in Table 1.

**Table 1:** Pearson correlation coefficients for personality (RST-PQ, N = 166).

	1 BAS-GDP	2 BAS-RI	3 BAS-RR	4 BAS-I	5 BIS	6 FFFS
1	1					
2	**0.51** ^**‡**^	1				
3	**0.37** ^**‡**^	**0.50** ^**‡**^	1			
4	0.033	**0.35** ^**‡**^	**0.42** ^**‡**^	1		
5	−0.12	**−0.23** ^•^	0.04	0.14	1	
6	0.06	−0.10	**0.20** ^•^	−0.08	**0.27** ^**†**^	1
Mean	20.8	19.0	28.6	18.4	53.0	25.7
STD	3.6	4.1	4.5	4.3	9.9	5.1
Range	9–28	9–28	15–39	8–31	33–82	14–39

Behavioral Approach System (BAS): Goal-Drive Persistence (BAS-GDP), Reward Interest (BAS-RI), Reward Reactivity (BAS-RR), and Impulsivity (BAS-RI); Behavioural Inhibition System (BIS) and Fight-Flight-Freeze System (FFFS).

Correlations between alpha asymmetry scores and personality measures were performed across the recording sites of interest for the Tot-Group, FF-Group, and FM-Group. The BAS-GDP, BAS-RI, BAS-RR, and BIS traits were all unrelated to alpha asymmetry scores of interest (see Table 2). As shown in this table, the bivariate relationships between the personality variables and asymmetry scores for central and parietal sites were also not significant (p > 0.10). Interesting, BAS-I in both the Tot-Group and FF-Group, but not in FM-Group, was significantly positively correlated with both frontopolar (Fp1, Fp2), frontal (F3, F4) alpha asymmetry scores. In addition, FFFS, but not BIS, was found significantly and negatively correlated with frontocentral (FC3, FC4) in both the Tot-Group and FF-Group, but not in FM-Group (Table 2). It is interesting to note that these significant associations still hold after partialling out the effect of experimenter’s gender in the Tot-Group, i.e., values of z, obtained to assess the significance of the difference between significant correlations, ranged from −0.11 to 0.01.

**Table 2:** Correlations of personality trais with resting alpha asymmetry in three groups of female participants: (1) total group (Tot-Group, N = 162) including two young experimenters of different genders; (2) subgroup with a female experimenter (FF-Group, N = 105); (3) subgroup with a male experimenter (FM-Group, N = 57).

	BAS-GDP	BAS-RI	BAS-RR	BAS-I	BIS	FFFS
**Tot-Group**						
Frontopolar Asymmetry	−0.07	0.08	0.13	0.39^‡^	0.09	−0.14
Frontal Asymmetry	−0.12	−0.02	0.07	0.33^‡^	0.11	−0.10
Frontocentral Asymmetry	−0.14	−0.10	−0.07	0.11	−0.01	−0.17*
Central Asymmetry	−0.13	−0.12	−0.04	0.13	0.00	−0.15
Parietal Asymmetry	−0.11	−0.12	−0.04	0.10	0.08	−0.04
**FF-Group**						
Frontopolar Asymmetry	−0.10	0.05	0.15	0.52^‡^	0.16	−0.15
Frontal Asymmetry	−0.13	−0.10	−0.02	0.45^‡^	0.15	−0.16
Frontocentral Asymmetry	−0.20	−0.16	−0.14	0.11	0.03	−0.25^•^
Central Asymmetry	−0.14	−0.15	−0.06	0.19	0.06	−0.17
Parietal Asymmetry	−0.07	−0.14	0.04	0.12	0.14	0.10
**FM-Group**						
Frontopolar Asymmetry	−0.01	0.18	0.05	0.13	−0.08	−0.11
Frontal Asymmetry	−0.03	0.12	0.20	0.17	−0.08	−0.01
Frontocentral Asymmetry	−0.12	−0.01	0.02	0.11	−0.06	−0.08
Central Asymmetry	−0.12	0.03	−0.02	0.05	−0.10	−0.12
Parietal Asymmetry	−0.21	−0.07	−0.24	0.03	−0.08	−0.21

^‡^p < 0.0001; ^•^p < 0.01; *p < 0.05.

To highlight which personality variables predicted resting alpha asymmetry scores, we performed three separate multiple regressions, each with frontopolar, frontal, and frontocentral alpha asymmetry scores as the dependent variable, wherein BAS-I, BAS-GDP, BAS-RI, BAS-RR, BIS and FFFS were entered into the model (see Table 3). BAS-I predicted higher relative left frontopolar activity in both the Tot-Group (F(6, 155) = 5.43, p < 0.0001, R^2^ = 0.17; β = 0.37, p < 0.0001) and the FF-Group (F(6, 98) = 6.85, p < 0.0001, R^2^ = 0.30; β = 0.51, p < 0.0001), whereas for the FM-Group, this relation failed to reach significance (F(6, 50) = 0.47, p > 0.05, R^2^ = 0.05; β = 0.02, p > 0.05). A similar regression was also significant for frontal asymmetry in both the Tot-Group (F(6, 155) = 4.34, p = 0.0004, R^2^ = 0.14; β = 0.35, p = 0.0002) and the FF-Group (F(6, 98) = 6.99, p < 0.0001, R^2^ = 0.30; β = 0.50, p < 0.0001) but not in the FM-Group (F(6, 50) = 0.58, p > 0.05, R^2^ = 0.06; β = 0.04, p > 0.05). In addition, FFFS scores were near the significance level as predictors of frontocentral asymmetry for the Tot-Group (F(6, 155) = 1.95, p = 0.07, R^2^ = 0.07; β = −0.15, p = 0.07) and were a significant predictor for the FF-Group (F(6, 98) = 2.21, p < 0.05, R^2^ = 0.12; β = −0.22, p = 0.037), but again, for the FM-Group, this relation was not significant (F(6, 50) = 0.34, p > 0.05, R^2^ = 0.04; β = −0.06, p > 0.05). In the above models, only BAS-I and the FFFS were significant predictors of alpha asymmetry scores (Table 3).

**Table 3:** Multiple regression of frontopolar (Fp1, Fp2), frontal (F3, F4) and frontocentral (FC3, FC4) asymmetries on RST-PQ traits.

	Tot-Group (N = 162)			FF-Group (N = 105)			FM-Group (N = 57)		
	β	*t*	*p*	β	*t*	*p*	β	*t*	*p*
**Frontopolar**									
**Asymmetry**									
BAS_GDP	−0.06	−0.68	0.50	−0.05	−0.49	0.63	−0.13	−0.78	0.44
BAS_RI	−0.03	−0.29	0.77	−0.08	−0.69	0.49	0.23	1.12	0.27
BAS_RR	0.04	0.37	0.71	0.02	0.15	0.88	0.01	0.04	0.96
BAS_I	**0.37**	**4.23**	**<0.0001**	**0.51**	**5.11**	**<0.0001**	0.02	0.13	0.89
BIS	0.06	0.71	0.48	0.05	0.51	0.61	0.01	0.03	0.97
FFFS	−0.13	−1.63	0.10	−0.13	−1.42	0.16	−0.07	−0.45	0.65
**Frontal**									
**Asymmetry**									
BAS_GDP	−0.07	−0.77	0.44	−0.06	−0.56	0.58	−0.15	−0.87	0.39
BAS_RI	−0.12	−1.11	0.27	−0.13	−1.10	0.27	0.07	0.37	0.72
BAS_RR	0.03	0.26	0.80	−0.13	−1.11	0.27	0.20	1.11	0.27
BAS_I	**0.35**	**3.87**	**0.0002**	**0.50**	**5.00**	**<0.0001**	0.04	0.22	0.83
BIS	0.06	0.71	0.48	0.12	1.31	0.19	−0.05	−0.3	0.77
FFFS	−0.11	−1.29	0.20	−0.13	−1.47	0.15	−0.02	−0.15	0.88
**Frontocentral**									
**Asymmetry**									
BAS_GDP	−0.09	−1.00	0.32	−0.07	−0.6	0.55	−0.13	−0.76	0.45
BAS_RI	−0.12	−1.07	0.29	−0.14	−1.07	0.29	−0.05	−0.23	0.82
BAS_RR	−0.01	−0.08	0.94	−0.06	−0.48	0.63	0.05	0.25	0.80
BAS_I	0.15	1.59	0.11	0.17	1.47	0.14	0.11	0.61	0.54
BIS	−0.02	−0.26	0.80	0.01	0.07	0.94	−0.06	−0.34	0.73
FFFS	−0.15	−1.82	0.07	−0.22	**−2.12**	**0.04**	**−0.06**	**−0.38**	0.70

## Discussion

Alpha asymmetry, calculated at conventional scalp sites, showed that higher BAS-I was highly related to greater relative left frontopolar and left-frontal activation. This relation was significant for both the total group, of which only approximately one-third of the participants had a male experimenter, and for the FF-Group, in which the experimenter was a young female. In addition, for the FF-Group, we also found a negative association of the FFFS trait with frontocentral asymmetry. This association for the total group of participants showed a trend towards significance (p = 0.07), whereas for the FM-Group, in which the experimenter was a young male, we failed to find any significant relationship between alpha asymmetry and personality measured by RST-PQ traits. Importantly, these results are the first to link BAS-I and FFFS measures of RST-PQ with greater resting left-frontal activity and right frontocentral activity, respectively. Additionally, our findings indicate that these links, obtained in female participants, are more robust when the experimenter is a young female. On the whole, the present findings support previous work establishing the connection between trait impulsivity (reduced regulatory control) and greater relative left-frontal activation^33,42,43^. The results also suggest that the active avoidance, as measured by FFFS, may be more closely tied to right-frontal activity than the superordinate BIS. These findings appear in line with those of Gray and McNaughton^3^, who indicated a substantial separation of FFFS/fear and BIS/anxiety processes. Nevertheless, the present findings contradict many of the previous reports that, despite evidence, found no link between the FFFS and higher resting relative right frontocentral activity^34^. These observations corroborate previous work^24,38,44^ questioning the link between Gray’s original conception of BIS as an avoidance system and greater right-frontal activity. Overall, our results corroborate the validity of biological models of approach-avoidance behaviour that focus primarily on the system of approach/BAS and avoidance/FFFS^15–22^ and indicate that, in previous work prior to the substantial revision of RST, the failures to replicate the link between active-avoidance motivation and greater resting right-frontal activity could be because the theoretical complexity of avoidance motivation may have confounded behavioural functioning of the FFFS (active avoidance/escape) or the BIS (conflict-related avoidance). Using the RST-PQ, which provides separate measures for the BIS and the FFFS, rather than supporting a single BIS scale, the current results indicate that the relationship between greater right-frontal activity and the FFFS scale may be driven by individual difference in pure avoidance/escape.

**Figure 1:**
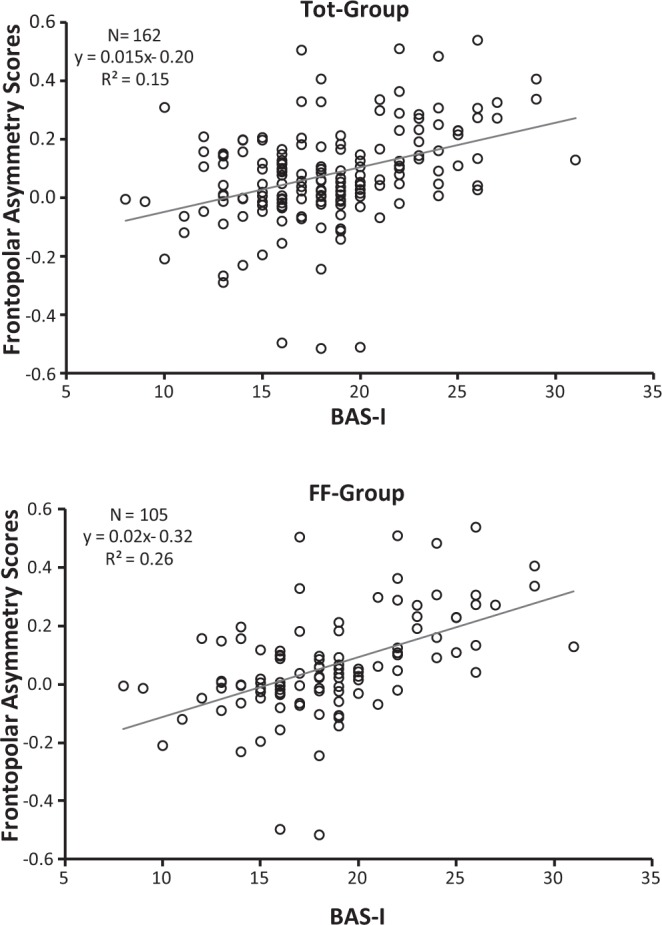
BAS-I Scores and Frontopolar Asymmetry Scores (Fp2–Fp1) in the total group (Tot-Group, upper scatterplot) and in the subgroup with a female experimenter (FF-Group, bottom scatterplot). Higher positive Frontopolar Asymmetry Scores indicate greater relative left frontopolar activity, vice versa, higher negative Frontopolar Asymmetry Scores indicate greater relative right frontopolar activity.

**Figure 2:**
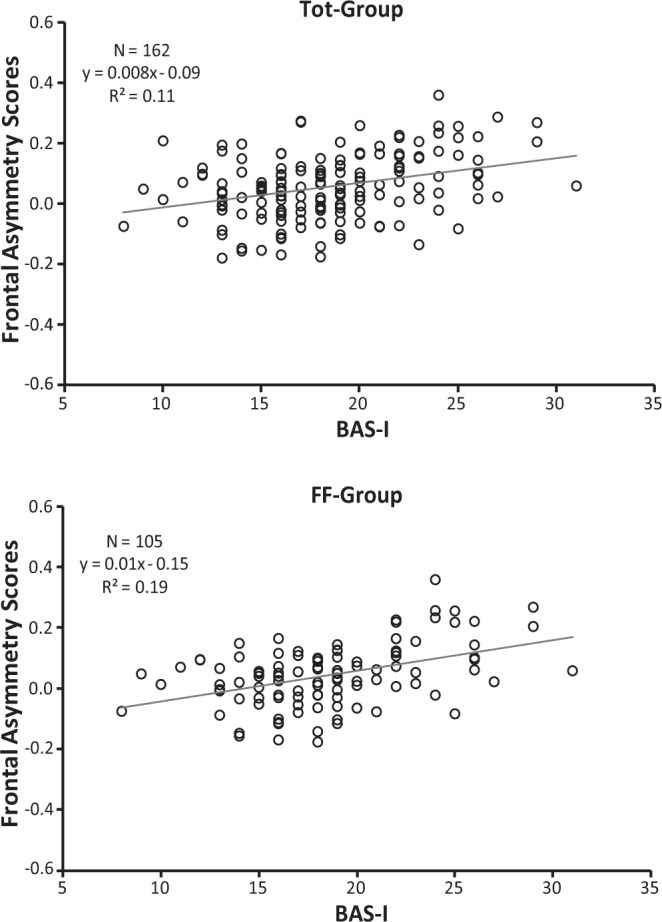
BAS-I Scores and Frontal Asymmetry Scores (F4–F3) in the total group (Tot-Group, upper scatterplot) and in the subgroup with a female experimenter (FF-Group, bottom scatterplot). Higher Frontal Asymmetry Scores indicate greater relative left frontal activity.

**Figure 3:**
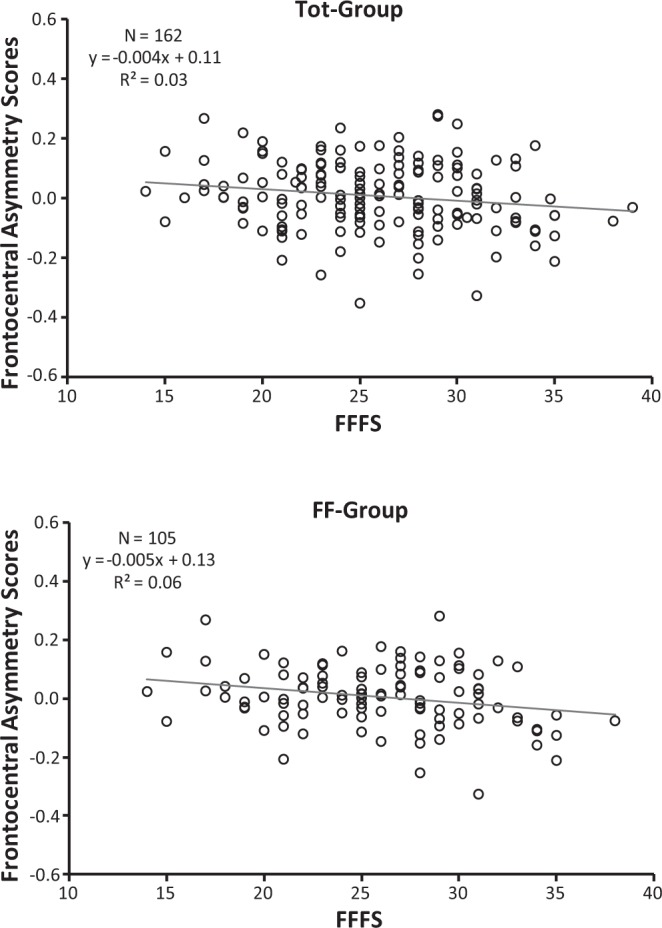
FFFS Scores and Frontocentral Asymmetry Scores (FC4-FC3) in the total group (Tot-Group, upper scatterplot) and in the subgroup with a female experimenter (FF-Group, bottom scatterplot). Smaller Frontal Asymmetry Scores indicate greater relative right frontal activity.

In terms of the influence of sex concordance between participants and experimenters in the relation of personality traits and EEG alpha asymmetry, our findings indicate that sex concordance may influence EEG resting activity during recording in the laboratory. However, the current results indicate that the association of the BAS-I trait to greater left frontopolar and frontal activity was sufficiently robust to be significant in the whole group of female participants (Tot-Group, Figs 1 and 2), and to a larger significance extent in the group in which the experimenter was a young woman (FF-Group, Figs 1 and 2). A similar consideration can be derived for the association of the FFFS and relative right hemisphere activation (i.e., the association was not significant in the presence of the opposite-sex experimenter; see Fig. 3). However, all these significant associations vanished for the group of participants in which the experimenter was a young male. It is nonetheless important to underline that we obtained significant personality-asymmetry associations in both the case of concordance in gender between participant and researcher and in the whole group that included two experimenters of different genders. We conclude that in the association of resting frontal activity and personality traits, much of the variance is a result of trait influences, and the remaining variance is at least partially dependent on features of the resting situation (i.e., in our case, if the gender of the young experimenter is the same or opposite to that of participants).This conclusion appears in line with previous findings obtained with 59 participants by Hagemann, *et al*.^45^, which showed that 60% of the variance in resting frontal activity is a result of trait influences, and 40% of the variance was due to occasion-specific fluctuations, but measurement errors were negligible^45,46^. However, it is important to note that we observed a robust personality-hemispheric asymmetry in the presence of the same-sex experimenter but not in the presence of opposite-sex experimenter, as observed by Kline and colleagues^47^ and the evidence provided in the review by Wacker, *et al*.^38^. According to these authors, individual differences in affective tendencies are dependent upon the interaction between the situational context and the innate capabilities of the individual^41^. This view has been supported by findings reported in a recent study, in which EEG asymmetry in male participants was correlated with their BAS scores only when the EEG recording was carried out by an attractive female experimenter, i.e., in a situation where approach motivation was highly salient but did not emerge without that contextual stimulus^38^. In contrast, the present study had only female participants, and the relation between personality and EEG alpha asymmetry was not significant in the case of opposite-sex experimenter, i.e., in a situation where approach motivation was prominent. This may have induced a potential greater embarrassment felt by female participants in presence of a male experimenter. In addition, since we did not collect sexual orientation of the participants we cannot generalize that all participants experienced an approach motivation, thus variability in sexual orientation may contribute to mask the effect. Considering that this state of embarrassment is variable across female participants, we think that much of the variance in resting frontal activity was more dependent on the extent to which each female participant perceived the young male experimenter as an attractive person. In this case, it may be that situational variables were particularly strong and may overwhelm the influence of a trait relationship with the resting EEG measure. This is not surprising, considering that nearly half of the variance on resting EEG asymmetry of the variance is due to state influences^45,48^.

In sum, results of the current study suggest that the FFFS trait appears to be related to greater right-frontal activity, although in the whole group that included fluctuations in resting alpha asymmetry caused by the influence of the experimenter’s opposite gender, the effect size of this relationship was quite small. We believe that this rare finding in the literature of resting asymmetry and the FFFS is important since it is the first to disclose a relation between this active avoidance trait and resting right-frontal activity. This result was obtained thanks to the new conceptualization of the revised RST as the RST-PQ, which provides separate measures of the BIS and the FFFS. In addition, the present results have shown a robust relationship between BAS-I and higher relative resting left-frontal activity, a finding that parallels and extends previous findings by Neal and Gable^33^ that relate greater trait impulsivity to less relative right-frontal activation.

One limitation of the current study is that only females were invited to participate and, thus, findings cannot be generalized to men. Additionally, we did not collect information about sexual orientation of the female participants in presence of an opposite-sex experimenter. This may have contributed to mask the relationship between personality and EEG-alpha asymmetry. Further studies are necessary that control for gender, sexual orientation, and multiple measurements of the interaction between participant and experimenter. Such studies may help researchers attain a better understanding of what traits contribute to resting frontal activity and, in turn, better understand the relationship between neural correlates and behaviour in regard to individual vulnerability to behavioural dysfunctions.

## Methods

### Participants

One hundred and sixty-nine right-handed female psychology students participated for course credit. Due to technical problems with EEG recordings, the data sets of seven participants could not be completed. The remaining 162 female participants defined the sample of the present study (18–34 yrs, M = 23.4, SD = 2.3). Hand preference was assessed using the Italian version of the Edinburgh Handedness Inventory^49^. Participants reported no history of neurological or psychiatric problems, and those who were in a menstrual period were invited for the EEG recordings between the 5th and 11th day after the onset of menses to avoid possible changes in EEG asymmetry linked to the menstrual period. Participants were seen individually in the lab and were informed about the nature of the study upon arrival. They gave informed consent prior to their inclusion in the study, which was approved by the ethics committee of the Department of Psychology in accordance with the Helsinki Declaration. Of the 162 participants, 105 females (19–34 yrs, M = 23.6, SD = 2.4) had a young female experimenter and 57 (18–34 yrs, M = 23.2, SD = 2.2) a young male experimenter.

### Materials

The participants completed the RST-PQ^35^. The RST-PQ consisted of 71 statements and measures three major systems: (1) the FFFS, related to fear; (2) the BIS, related to anxiety; and (3) the BAS, measuring four behavioural approach facets: BAS-GDP (e.g., “I often overcome hurdles to achieve my ambitions”); BAS-RI (e.g., “I am always finding new and interesting things to do”); BAS-RR (e.g., “I am especially sensitive to reward”); BAS-I (e.g., “I sometimes cannot stop myself talking when I know I should keep my mouth closed”). Cronbach’s α values for the BIS and the FFFS, respectively, were 0.86 and 0.78. Alpha values for the BAS-GDP, BAS-RI, BAS-RR, and BAS-I were, respectively, 0.87, 0.75, 0.78, and 0.76.

### Procedure

Each participant was seated in an electrically shielded EEG booth, and electrodes were applied to measure EOG and EEG. Participants accomplished 8 min of baseline resting EEG recording, half with eyes open and half with eyes closed, in a counterbalanced order across participants. They were instructed to remain still and to inhibit blinks or eye movements during each recording period. During the eyes-open condition, participants had to fixate on a white cross presented in the centre of a computer monitor that was 1.2 m distant from their head.

### EEG recording and processing

Scalp EEG was recorded using a 32-channel tin electrode stretch Lycra cap (Electro-Caps, Eaton, OH, USA). Electrode placement was based on the 10–20 system, with a ground electrode mounted between FPz and Fz. Prior to the placement of electrodes, the expected electrode sites on the participant’s scalp and face were cleaned with an alcohol solution. Electrode impedances were kept under 5 kΩ, with homologous sites held within a difference of 1 kΩ. EEG activity was referenced online to the left earlobe. Bipolar horizontal and vertical EOG were recorded, respectively, from the epicanthus of the right and the left eye and from the supra- and infra-orbital positions of the right eye using standard tin electrodes. EEG was recorded using a 40-channel NuAmp acquisition system (Neuroscan Inc., Herdon, VA, USA) set in DC mode, with a gain of 200 (100 for eye channels), a bandpass of 0.01–100 Hz (Butterworth zero phase filter with 24 dB/octave roll off), and an online notch filter at 50 Hz. The signals were sampled at 1000 Hz. After data acquisition was accomplished, the continuous EOG and EEG recordings were visually inspected, and each portion of the EEG data showing ocular, muscular, or technical artefacts in any channel was rejected for this and all simultaneously recorded channels^50,51^. The ocular correction procedure on EEG data was performed using the method by Gratton, *et al*.^52^. After ocular correction, data were again visually inspected for appropriate removal and correction of artefacts. All epochs were extracted through a Hamming window, which was specified to diminish the signal on 20% at the beginning and on 20% at the end of the epoch, to prevent spurious estimates of spectral power. After artefact detection, all available artefact-free EEG epochs (on average, 355.7 epochs across subjects, SD = 61.1; approximately 6 min of EEG recording) were extracted from both the eyes-closed and eyes-open trials and subjected to conventional spectral analyses in accordance with Sutton and Davidson^14^ method.

Power spectra were calculated using Fast Fourier Transform (FFT). Prior to spectral analysis, an off-line linked-ears (A1 + A2) reference was computed to facilitate comparisons with previous work e.g.^14^. Each consecutive epoch overlapped by 50%.

The band of interest was the conventional alpha band (8–13 Hz), and power values were averaged across all epochs. Power values were natural-logarithm (ln) transformed to normalize the data^53^. Following previous research, alpha asymmetry measures were calculated as the right minus left difference of ln power values of homologous scalp sites of interest, such that higher scores indicated greater left-sided cortical activity. The following five alpha asymmetry scores were calculated: frontopolar (Fp2–Fp1), frontal (F4–F3), frontocentral (FC4–FC3), central (C4–C3), and parietal (P4–P3). These measures served for correlation analyses with personality measures. The artefact screening, re-referencing, and spectral analysis were performed with Brain Vision Analyzer 2.0 (Brain Product GmbH, Gilching, Germany), and further computation of asymmetry measures was performed using SAS version 9.4.

To test the influence of the experimenter’s gender on EEG alpha asymmetry, separate correlation analyses were performed in the following three groups of participants: (1) total group (N = 162), wherein 57 participants had a male experimenter (Tot-Group); (2) group (N = 105) with a female experimenter (FF-Group); (3) group (N = 57) with a male experimenter (FM-Group).
